# CD36 as a Context-Dependent Regulator of Metabolic Switching in Acute and Chronic Hypoxia

**DOI:** 10.3390/biom16071018

**Published:** 2026-07-12

**Authors:** Mihaela R. Popescu, Anca M. Panaitescu, Laura Cristina Ceafalan, Mihail Eugen Hinescu

**Affiliations:** 1 Department of Cardiology, Elias Emergency University Hospital, University of Medicine and Pharmacy “Carol Davila”, 020021 Bucharest, Romania; 2 Department of Obstetrics and Gynecology, Filantropia Clinical Hospital, University of Medicine and Pharmacy “Carol Davila”, 011132 Bucharest, Romania; 3 Department of Cellular and Molecular Biology and Histology, University of Medicine and Pharmacy “Carol Davila”, 011132 Bucharest, Romania; 4 Victor Babes National Institute of Pathology, 050096 Bucharest, Romania

**Keywords:** metabolic modulator, lipid metabolism, ischemia–reperfusion injury, HIF-1α, macrophages, inflammation, tumorigenesis

## Abstract

CD36 is a multifunctional scavenger receptor involved in long-chain fatty acid (LCFA) uptake, binding of oxidized lipids, and interactions with extracellular matrix proteins such as thrombospondin-1. Through association with Src family kinases, integrins, and adaptor proteins, it also modulates signaling, migration, inflammation, angiogenesis, and phagocytosis. Hypoxia, a common feature of solid tumors, inflamed tissues, and ischemic organs, remodels CD36 expression, localization, and function through hypoxia-inducible factor (HIF) signaling and stress-activated kinases. These effects change cellular metabolism, intercellular lipid trafficking, and cell behavior (migration, phagocytosis, angiogenesis, immune phenotype) in a manner that is highly dependent on tissue type, duration of hypoxia, and metabolic context, with important implications for disease progression. In acute hypoxia, CD36 regulation often contributes to rapid metabolic adaptation, whereas in chronic hypoxia, it may promote sustained lipid accumulation, inflammation, maladaptive remodeling, or tumor progression. In this review, we aim to highlight the regulation and function of CD36 in hypoxia in different tissues, conditions, and metabolic states, emphasizing the distinct roles of CD36 in acute versus chronic hypoxia and its potential therapeutic implications. For example, hypoxia typically downregulates CD36 in ischemic cardiomyocytes to limit lipotoxic fatty acid influx, whereas in hepatocytes, adipocytes, and tumor-associated macrophages, it upregulates CD36-mediated lipid uptake to sustain steatotic, inflammatory, or protumorigenic metabolism, illustrating the tissue-specific nature of this regulation.

## 1. Introduction

CD36 is a multifunctional scavenger receptor with several alternative names: fatty acid translocase (FAT), scavenger receptor B2 (SR-B2), and platelet membrane protein IV (GP IV) [1]. Depending on tissue type, metabolic conditions (e.g., hypoxia), and posttranslational processing (e.g., phosphorylation), CD36 performs different functions. CD36 is involved in many physiologic processes, such as angiogenesis, cell adhesion, and lipid metabolism [2]. CD36 is expressed by macrophages [3], adipocytes [1,3], cardiac muscle cells [4], renal tubular epithelial cells [5], liver cells [6], platelets [7,8], endothelial cells [9], and circulating endothelial cells (CECs) [10].

Dysregulation of CD36 expression has been linked to multiple pathological processes ranging from atherosclerosis to cancer [1,11]. Hypoxia initiates a cascade of molecular and cellular events that induce initial tissue remodeling, angiogenesis, and metabolic adaptation. These compensatory mechanisms activate metabolic pathways and adaptive responses to ischemic insult. However, prolonged hypoxia can lead to tissue damage in conditions such as ischemic heart disease, myocardial infarction, atherosclerosis, stroke, tumor development, or placental insufficiency, and these compensatory mechanisms become clinically relevant and sometimes deleterious.

The risk for atherosclerosis is associated with both high and low CD36 levels. While CD36 overexpression drives inflammation, endothelial dysfunction, foam cell formation, and thrombosis, CD36 deficiency is linked to dyslipidemia, impaired oral fat tolerance, and increased susceptibility to insulin resistance. This raises the question of whether there is an optimal CD36 level that might be reached through modulation in the posttranslational stage.

Recent studies have shown that, through mechanisms involving hypoxia-inducible factors (HIFs), transcriptional regulators, and posttranslational modifications, hypoxia alters CD36 expression and function. However, the effects of hypoxia on CD36 may differ substantially between acute and chronic hypoxic exposure, as well as during physiological adaptation and disease states. These changes assist in metabolic adaptation but may also promote pathological processes such as lipid accumulation, inflammation, and tissue remodeling.

Understanding how hypoxia regulates CD36 throughout various tissues and disease contexts is therefore essential for illuminating its role in metabolic and inflammatory disorders.

Therefore, CD36 is a promising therapeutic target for diseases involving tissue hypoxia or a hypoxia-like environment (e.g., tumorigenesis) or for patients at risk for premature vascular aging [12], with classical cardiovascular risk factors.

We searched PubMed, Scopus, and Web of Science from database inception through January 2026 using combinations of the following search terms: CD36, FAT, SR-B2, scavenger receptor B2, fatty acid translocase, hypoxia, and specific disease terms relevant to each tissue section (e.g., myocardial infarction, glioblastoma, and preeclampsia). We prioritized primary research articles with mechanistic content but also included convincing reviews, relevant clinical studies, and seminal earlier work retrieved through reference tracking. Preference was given to studies that explicitly reported the duration of hypoxic exposure, which allowed classification within the acute/subacute/chronic framework (defined operationally in Section 4) used throughout this review. Non-English-language publications, conference abstracts without subsequent full publication, and preprints without peer review were excluded. This review follows a narrative synthesis approach rather than a systematic review protocol, and the search strategy is reported here for transparency rather than as a formal PRISMA exercise.

This review aims to summarize the roles of CD36 in hypoxic environments and discuss how hypoxia-induced signaling pathways modulate CD36 expression and activity across diverse tissue and disease contexts. Particular attention is given to the mechanisms involved in acute versus chronic hypoxia and their effects on metabolic remodeling, inflammation, and disease progression. Finally, we emphasize the potential of CD36 as a therapeutic target in disorders characterized by hypoxia-driven metabolic dysfunction, including cardiovascular disease, metabolic liver disease, cancer, and neurovascular injury.

Across all the tissue and disease contexts reviewed below, hypoxia-driven CD36 regulation can be understood within a single conceptual framework of metabolic switching. An initial, largely trafficking-driven adaptive phase, in which acute energy stress sensed via AMP-activated protein kinase (AMPK) and rapid changes in CD36 membrane localization limit fatty acid influx when mitochondrial oxidative capacity is acutely constrained, is followed, as hypoxia persists, by a transcriptional phase in which a shift in the balance of HIF-1α- and HIF-2α-driven programs progressively increases CD36-mediated lipid influx beyond mitochondrial oxidative capacity, driving the transition from adaptive substrate flexibility to maladaptive lipid accumulation, inflammation, and tissue remodeling. This framework, developed mechanistically in Section 4 and applied to each tissue and disease context in Section 5, provides a common scaffold for reconciling the apparently contradictory roles that CD36 plays in hypoxia.

## 2. CD36 Biology: Ligands, Signaling Partners, and Trafficking

### 2.1. Ligands and Functional Diversity

One of the defining features of CD36 is its ability to bind structurally diverse ligands, which underlies its broad functional repertoire.

Long-chain fatty acids (LCFAs): CD36 facilitates the cellular uptake of fatty acids, particularly in tissues with high oxidative demand, such as the heart and skeletal muscle. Depending on the metabolic context, this function may support energy production or contribute to lipid accumulation and lipotoxicity [13].

Oxidized lipoproteins (e.g., oxLDL): Binding of oxidized lipids links CD36 to inflammatory signaling, foam cell formation, and atherosclerosis. In macrophages, CD36-mediated oxLDL uptake is a key step in foam cell development [14].

Thrombospondin-1 (TSP-1) and related matricellular proteins: Interaction with TSP-1 promotes antiangiogenic signaling, endothelial apoptosis, and the modulation of immune responses, often counteracting VEGF-mediated pathways [15].

Because these ligands activate distinct downstream pathways, CD36 acts as an integrator of metabolic and inflammatory signals. The relative abundance of each ligand in a given microenvironment strongly influences whether CD36 activity is adaptive or pathogenic.

The effects associated with each ligand are presented in Table 1.

### 2.2. Signaling Partners and Downstream Pathways

CD36 participates in multiple signaling pathways through interactions with membrane-associated proteins and intracellular adaptors. It forms complexes with Src family kinases, integrins, and other scaffolding proteins, enabling the activation of downstream signaling cascades [14].

CD36 signaling has been linked to the activation of the MAPK and NF-κB pathways, contributing to inflammatory responses; the regulation of reactive oxygen species (ROS) production; the modulation of cell migration and adhesion; the control of phagocytosis and efferocytosis in macrophages [17]; and the regulation of angiogenic and antiangiogenic signaling depending on ligands [14].

CD36 lacks intrinsic catalytic activity and signals through the recruitment of cytoplasmic partners to its short C-terminal tail. The principal proximal signaling partners are the Src family kinases Fyn and Lyn, which couple ligand-engaged CD36 to downstream MAP kinase (JNK and p38) and PI3K–Akt cascades and, in some contexts, to the focal-adhesion kinase–integrin axis. Different ligand classes preferentially engage different partners: oxidized lipids drive Fyn/Vav-dependent inflammatory and foam-cell-formation signaling; thrombospondin-1 binding involves a Fyn → p38 → caspase-3 antiangiogenic axis; and long-chain fatty acid binding activates trafficking-related signaling that coordinates membrane CD36 with intracellular fatty acid handling. These signaling interactions help explain why CD36 can mediate not only substrate uptake but also inflammatory, angiogenic, and phagocytic responses under hypoxic stress.

### 2.3. Regulation and Modulation of CD36

**Figure 1:**
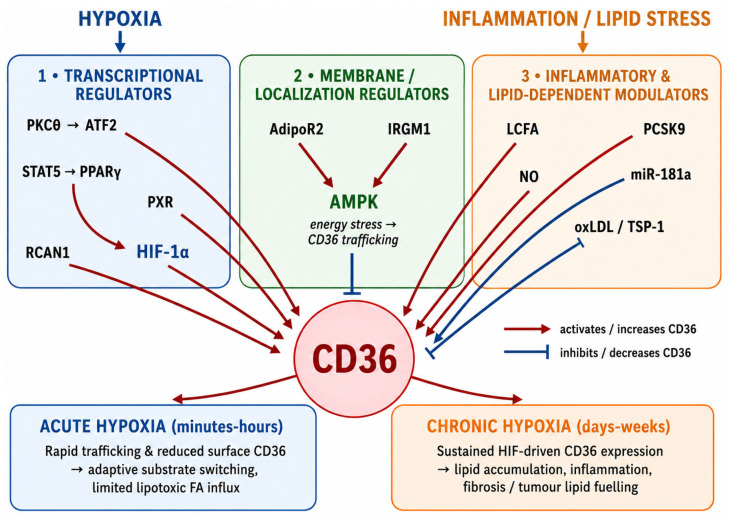
Endogenous regulation of CD36. ATF2—activating transcription factor 2; AdipoR2—adiponectin receptor 2; AMPK—AMP-activated protein kinase; IRGM1—immune-related GTPase M; HIF-1α—hypoxia-inducible factor-1α; LCFA—long-chain fatty acids; oxLDL—oxidized low-density lipoprotein; miR-181a—microRNA-181a; NO—nitric oxide; PCSK9—proprotein convertase subtilisin/kexin type 9; PPAR-γ—peroxisome proliferator-activated receptors-γ; PKCθ—protein kinase C-theta; PXR—pregnane X; TSP-1—thrombospondin-1.

CD36 activity is regulated by a wide array of molecular pathways that may be activated under pathological conditions such as hypoxia, diabetes, dyslipidemia, and inflammation (Figure 1). For clarity, these pathways can be organized into three conceptual clusters: (1) transcriptional regulators, (2) membrane/localization regulators, and (3) inflammatory and lipid-dependent modulators.

#### 2.3.1. Transcriptional Regulators

Transcriptional control of CD36 involves promoter selection, transcription factors, and nuclear receptor signaling. CD36 has more than five spliced variants that depend on different promoters. For example, the V1/V3 promoter is responsible for endothelial CD36 expression and depends on peroxisome proliferator-activated receptor γ (PPAR-γ) [18]. Signal transducer and activator of transcription 5 (STAT 5) activates PPARγ, thereby increasing CD36 expression [1]. HIF-1α, although strongly linked to proinflammatory signaling, also regulates CD36 expression [8]. Protein kinase Cθ (PKCθ) promotes CD36 expression by activating transcription factor 2 (ATF2) [2,3]. The pregnane X receptor (PXR) increases CD36 expression and lipid accumulation in macrophages [4], whereas regulator of calcineurin 1 (RCAN1) promotes CD36 expression, enhances lipid accumulation, and contributes to atherosclerosis [5].

#### 2.3.2. Membrane/Localization Regulators

Beyond transcriptional control, CD36 activity is also shaped by intracellular trafficking and membrane localization. Adiponectin receptor 2 (AdipoR2) increases both the expression and membrane localization of CD36, particularly in skeletal muscle [6]. A further mechanism relevant to foam cell formation is IRGM1-regulated CD36 internalization, which increases oxLDL uptake [7]. These pathways indicate that the functional impact of CD36 depends not only on how much the receptor is expressed but also on where the receptor is localized and how efficiently it is trafficked. This distinction between total and membrane-localized CD36 is methodologically important and is discussed further in Section 7.

#### 2.3.3. Inflammatory and Lipid-Dependent Modulators

Lipid ligands and inflammatory mediators directly modulate CD36 signaling and its downstream consequences. Fatty acids (FAs) bind to CD36 and promote CD36-mediated uptake of ox-LDL by macrophages, endothelial cells, and smooth muscle cells [19,20]. Nitric oxide (NO) is known to downregulate CD36 expression during inflammatory processes [21], most likely by decreasing PPAR-γ activity. MiR-181a overexpression reverses the ox-LDL-induced upregulation of CD36 expression in macrophages and reduces foam cell formation [8]. Proprotein convertase subtilisin/kexin type 9 (PCSK9), in addition to its regulatory role in circulating lipid levels, can also activate platelets and promote aggregation and thrombotic events by binding to platelet CD36 [22]. Aspirin and PCSK9 inhibitors such as evolocumab abolish this effect [23]. Given the observations linked to its effects on CD36, macrophages, and phagocytosis, CD36 may also play a role in the long-term resolution of inflammation [24].

### 2.4. Pharmacological Modulation of CD36

Because CD36 is involved in the convergence of lipid uptake, inflammatory signaling, and foam-cell formation, it is an attractive pharmacological target, and a growing number of drugs and bioactive compounds have been shown to modulate its expression or activity. Pharmacological modulation of CD36 represents a potential therapeutic strategy to inhibit its activity and mitigate its role in atherosclerosis (Table 2).

## 3. Mechanisms Linking Hypoxia to CD36

Hypoxia may have several effects on CD36, including transcriptional regulation, energy-sensing pathways, membrane trafficking, posttranslational modifications (PTMs), and ligand availability. In acute hypoxia, rapid signaling and transporter redistribution are prevalent, whereas in chronic hypoxia, sustained transcriptional and posttranslational programs become increasingly important.

**Figure 2:**
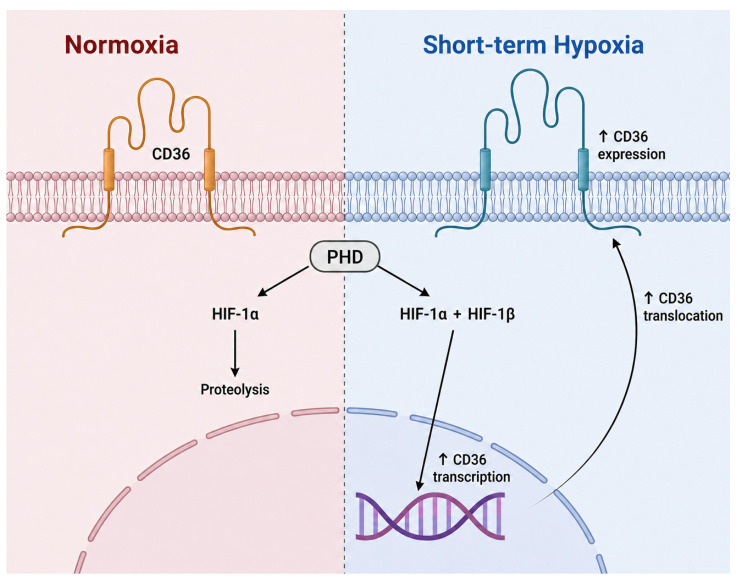
The impact of short-term (acute) hypoxia and HIF on CD36 expression. PHD—prolyl hydroxylase domain enzymes; HIF—hypoxia-inducible factor.

Increased cytoplasmic Ca^2+^ levels and hypoxia-inducible transcription factors (HIFs) are considered important effectors of hypoxic remodeling [21,24,27]. For example, HIF-1α has been repeatedly connected to CD36 in hypoxic and inflammatory environments, leading to CD36 transcription (Figure 2) [21,24]. However, this observation is valid only for short-term hypoxia, as long-term hypoxia appears to have the opposite effect on CD36 expression [28].

### 3.1. HIF-Dependent Transcription

Hypoxia-inducible factors (HIFs) are central mediators of the cellular response to low oxygen tension. Under normoxia, HIF-α subunits are hydroxylated by prolyl hydroxylase domain enzymes (PHDs) and targeted for proteasomal degradation. Under hypoxic conditions, hydroxylation is reduced, allowing HIF-α stabilization, nuclear translocation, dimerization with HIF-β, and transcriptional activation of genes involved in metabolism, angiogenesis, survival, and inflammation [27,29] (Figure 2). Activation of HIF signaling was also linked to CD36 regulation. However, this regulation depends on the tissue and isoform. For example, HIF-2α has been shown to increase CD36 expression and lipid accumulation in hepatocytes under hypoxic conditions, thereby promoting steatotic responses [28]. In contrast, HIF-1-dependent pathways are associated with adaptive responses that limit excessive lipid accumulation in some chronic liver disease models [30].

Taken together, HIF signaling links oxygen availability to CD36 expression, but the downstream consequences depend on the isoform and tissue context.

### 3.2. AMPK and Energy Stress

AMP-activated protein kinase (AMPK) is a key sensor of cellular energy stress. It is rapidly activated when ATP levels decrease and AMP/ADP levels increase. Because hypoxia limits mitochondrial oxidative phosphorylation, AMPK becomes an important early regulator of metabolic adaptation under low-oxygen conditions, promoting pathways that maintain ATP production [31].

AMPK likely contributes to the early metabolic context and rapid adjustment of substrate preference, in which CD36 redistribution becomes functionally important. Thus, metabolism shifts from fatty acid oxidation toward glucose utilization, a response that is often associated with changes in CD36 distribution and fatty acid uptake. A reduction in CD36 surface expression may help limit fatty acid influx when mitochondrial oxidative capacity is constrained, thereby reducing the formation of lipotoxic intermediates and supporting short-term survival [8]. This is highly relevant in ischemia–reperfusion injury, where excessive fatty acid delivery during impaired oxidation may aggravate metabolic stress.

A central effector that links HIF-1α stabilization to the metabolic switch is pyruvate dehydrogenase kinase 1 (PDK1). HIF-1α directly transactivates PDK1, which phosphorylates and inactivates the pyruvate dehydrogenase (PDH) complex, blocking pyruvate entry into the tricarboxylic acid cycle and actively downregulating mitochondrial oxygen consumption and ROS production [32,33]. This PDH block is the biochemical basis of the hypoxic, Warburg-like shift toward glycolysis, and it directly determines the functional consequence of CD36 activity: When mitochondrial fatty acid oxidation (FAO) is constrained, continued CD36-mediated fatty acid influx is diverted toward storage in perilipin-2 (PLIN2)-coated lipid droplets and toward incompletely oxidized intermediates, such as diacylglycerols, ceramides, and acylcarnitines, that promote insulin resistance, mitochondrial stress, and lipotoxicity [34]. This framework clarifies why the acute downregulation of surface CD36, by limiting fatty acid influx while PDH is inhibited, is metabolically protective, whereas sustained CD36-mediated influx under chronic hypoxia becomes maladaptive.

### 3.3. Trafficking and Membrane Localization

CD36 function depends strongly on its subcellular localization rather than its total protein expression level. The amount of CD36 present at the plasma membrane is a major determinant of fatty acid uptake and ligand interactions. One of the most important short-term mechanisms regulating CD36 during hypoxia is intracellular trafficking between endosomal compartments and the cell surface. The endosomal/sarcolemmal recycling of CD36 has been comprehensively reviewed in cardiac muscle [35], where it has been established as the principal determinant of cellular fatty acid uptake under physiological and pathological conditions. This distinction is especially important when interpreting acute hypoxia studies, in which rapid changes in membrane localization may dominate before transcriptional responses become detectable [24,29,34,36,37] (Table 3).

### 3.4. Posttranslational Modifications (PTMs) and the Ligand Environment

Posttranslational modifications (PTMs) are particularly important because they can alter receptor activity, localization, stability, and signaling without necessarily changing total gene expression. An emerging additional layer of regulation involves the recruitment of palmitoylated CD36 to lipid-raft microdomains by acid sphingomyelinase, which clusters CD36 with associated signaling partners and amplifies its lipid-uptake activity [49]. This mechanism connects CD36 palmitoylation, membrane microdomain organization, and ligand-induced signaling in a way that may be particularly relevant under inflammatory and hypoxic conditions, where both sphingolipid metabolism and CD36 palmitoylation are altered.

Among the PTMs relevant to CD36, palmitoylation has attracted attention because it influences membrane association and receptor trafficking [50]. Other modifications, including phosphorylation, ubiquitination, and redox-sensitive changes, may also modulate CD36 behavior directly or indirectly through interactions with other proteins. As a recent example, O-GlcNAcylation of CD36 has been shown to modulate its function in hypoxia/reoxygenation-injured cardiomyocytes, protecting against myocardial ischemia–reperfusion injury [51].

Ligand availability is also important. CD36 binds structurally and functionally diverse ligands, including long-chain fatty acids, oxidized LDL, thrombospondin-1, and oxidized phospholipids (Table 2). The dominant ligand present in a given hypoxic microenvironment can influence whether CD36 promotes adaptive substrate uptake, inflammatory signaling, antiangiogenic responses, or foam cell formation. Under lipid-rich and inflammatory conditions, oxidized lipids may amplify macrophage lipid loading and vascular injury [19,20].

Exposures of approximately 24 h are considered subacute and may involve both early adaptive responses and transcriptional regulation; they are grouped here with chronic hypoxia for simplicity.

## 4. CD36 in Acute vs. Chronic Hypoxia

**Figure 3:**
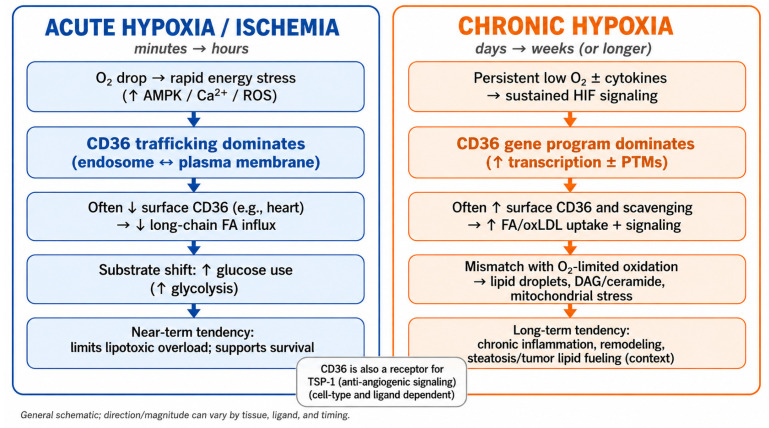
General mechanistic aspects of acute versus chronic CD36 activity. Color schematic summarizing typical dominant mechanisms: acute hypoxia is often trafficking-driven, whereas chronic hypoxia is often driven by gene programs and durable localization changes (cell-type-dependent). AMPK—AMP-activated protein kinase; DAG—diacylglycerol; FA—fatty acid; HIF—hypoxia-inducible factor; I/R—ischemia/reperfusion; LOX-1—lectin-like oxidized LDL receptor-1; MI—myocardial infarction; NAFLD—non-alcoholic fatty liver disease; OSA—obstructive sleep apnea; oxLDL—oxidized low-density lipoprotein; PAH—pulmonary arterial hypertension; PTM—post-translational modification; ROS—reactive oxygen species; TSP-1—thrombospondin-1.

In acute hypoxia, which starts from a few minutes to hours, the main action of CD36 is to facilitate rapid trafficking to quickly match lipid uptake to a suddenly limited oxidative capacity. In chronic hypoxia, which lasts from days to weeks, the effects of CD36 are more often driven by gene programs and durable changes in localization (and sometimes posttranslational modifications (PTMs) such as palmitoylation), which can reshape lipid handling long-term for better adaptation or lipotoxic remodeling. In some in vitro studies, acute hypoxia might extend to 24–48 h (which is better thought of as subacute, already involving transcriptional programs) (Figure 3) [52].

For clarity, throughout this review, we adopt the following operational definitions, in line with the dominant usage in the experimental literature: **acute hypoxia** denotes exposures of minutes to a few hours (typically ≤ 6 h in vitro, or the early reperfusion window in vivo), in which trafficking and posttranslational responses dominate; **subacute hypoxia** denotes exposures of approximately 24–48 h, in which transcriptional programs (HIF-1α-driven and increasingly HIF-2α-driven) become detectable and overlap with persistent trafficking responses—this window represents the transition zone from adaptive to maladaptive remodeling; and **chronic hypoxia** denotes exposures of days to weeks, dominated by sustained transcriptional and palmitoylation-driven programs. The oxygen levels in the cited studies span 0.1–5% O_2_ in vitro (typically 1% O_2_) and physiologically measured tissue pO_2_ in vivo (single-digit mmHg in hypoxic microenvironments such as solid tumors and atherosclerotic plaques). The distinction between hypoxia and ischemia is also important and is not always made explicit in the primary literature: ischemia involves restriction of both oxygen and substrate supply together with impaired clearance of metabolic byproducts, whereas isolated hypoxia (as modeled in most in vitro systems) leaves the substrate supply intact. Where the cited studies use ischemia–reperfusion models, this is indicated, and we note the caveats introduced in Section 7.

**Figure 4:**
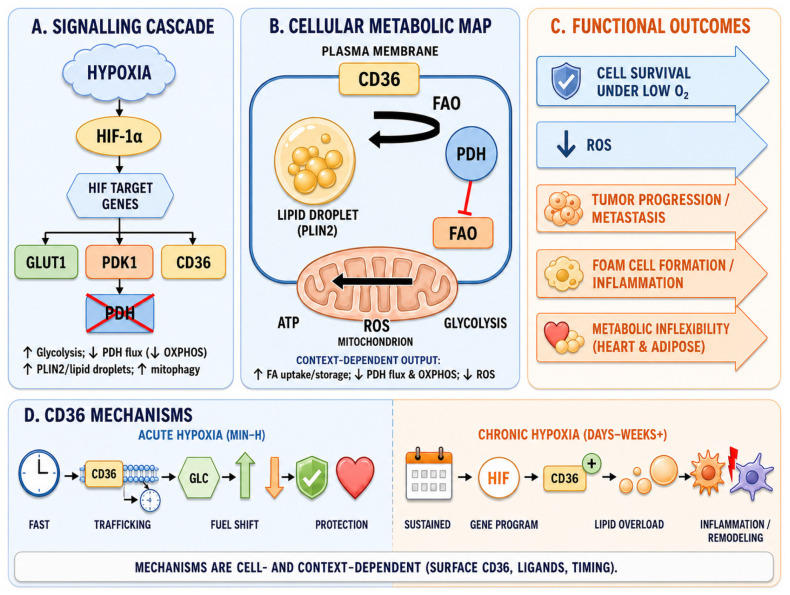
HIF–CD36 axis in hypoxic metabolic rewiring and time-dependent CD36 mechanisms in acute (in pink) versus chronic (in yellow) hypoxia: (**A**) HIF-1α–driven transcriptional response, including PDK1 induction and the glycolytic switch. (**B**) Cellular metabolic map: CD36-mediated long-chain fatty acid uptake, which is oriented toward PLIN2-coated lipid droplets or mitochondrial fatty acid oxidation; the effects of PDH inhibition on the glycolysis/oxidation balance and on ROS levels. (**C**) Functional outcomes: enhanced survival under low O_2_; context-dependent pathology, including tumor progression/metastasis and foam-cell formation/inflammation. (**D**) Acute (minutes–hours) versus chronic (days–weeks+) hypoxia: acute, trafficking-driven regulation of CD36, with short-term protective effects in some tissues; chronic, sustained HIF-driven programs that promote CD36-mediated lipid influx and accumulation in representative tissue contexts. ATP—adenosine triphosphate; CD36—cluster of differentiation 36; FA—fatty acid; FAO—fatty acid oxidation; GLUT1—glucose transporter 1 (SLC2A1); GLU—glucose; HIF—hypoxia-inducible factor; HIF-1α—hypoxia-inducible factor-1 alpha; O_2_—oxygen; PDH—pyruvate dehydrogenase; PDK1—pyruvate dehydrogenase kinase 1; PLIN2—perilipin 2.

By limiting FA influx, acute ischemia can protect the myocardium [34,52] (Figure 4). Chronic hypoxia induces CD36-driven lipid uptake and can become maladaptive (steatosis, remodeling, and tumor lipid fueling) [28]. CD36 localization at the plasma membrane often indicates increased lipid internalization, whereas changes in palmitoylation/trafficking can reprogram its net effect without substantial changes in total expression. Integrating these mechanisms, CD36 regulation across the metabolic switch can be summarized as a three-phase, tissue-dependent continuum (Figure 4D). In the acute phase (minutes–hours), AMPK activation and rapid CD36 internalization reduce fatty acid influx, whereas glucose transporter 1 and 4 (GLUT1/GLUT4) upregulation and HIF-1α–PDK1–PDH signaling favor glycolytic ATP production; this phase is predominantly adaptive, limiting the formation of lipotoxic intermediates when oxidative capacity is acutely constrained. In the subacute-to-chronic transition (hours–days), transcriptional and posttranslational programs become dominant: HIF-2α- and Nrf2-dependent signaling increases CD36 expression and lipid-droplet formation [28,53], and palmitoylation stabilizes CD36 at the plasma membrane largely independently of total protein levels [50]. In sustained chronic hypoxia (days–weeks), continued CD36-mediated lipid influx that outpaces a PDH-restricted mitochondrial compartment drives lipid accumulation, ROS production, and inflammatory signaling, which further stabilize HIF and reinforce lipid loading. Therefore, whether the net outcome is adaptive remodeling or lipotoxic and protumorigenic pathology depends on the balance between HIF-1α activity and HIF-2α activity, the prevailing ligand environment, and the metabolic and inflammatory background (e.g., diabetes and dyslipidemia). This continuum explains why CD36 appears to exert opposite effects across the tissues discussed below rather than behaving paradoxically.

The temporal transition between these phases is not merely descriptive but has a defined molecular basis in the differential regulation of the two principal HIF-α isoforms. HIF-1α protein is stabilized within minutes of the onset of hypoxia but is subject to strong negative feedback: prolyl hydroxylase domain protein 3 (PHD3) and factor-inhibiting HIF (FIH) are themselves HIF-1α target genes, and their reaccumulation during sustained hypoxia progressively decreases HIF-1α transactivation such that the HIF-1α response is inherently self-limiting over a period of hours. HIF-2α, by contrast, is stabilized more slowly, is less sensitive to FIH-mediated repression, and lacks an equivalent negative-feedback circuit; thus, HIF-2α accumulates progressively and becomes predominant in the transcriptional response once hypoxia is sustained beyond approximately 24–48 h; for this reason, HIF-1α is generally considered to drive the acute hypoxic response, whereas HIF-2α drives the chronic response [54]. Because CD36 is a direct or indirect target of both isoforms in a tissue-dependent manner (Section 3.1), this HIF-1α-to-HIF-2α handover provides a concrete molecular trigger for the shift from acute, trafficking-dominated CD36 regulation toward chronic, transcriptionally driven CD36 upregulation, rather than the transition being simply a matter of elapsed time. Reinforcing feed-forward loops, including HIF-2α-driven lipid accumulation itself, promotes further ROS generation and HIF stabilization, together with chromatin-level changes that consolidate transcriptional programs during sustained hypoxia, further stabilizing the chronic, maladaptive state once it is established.

**Figure 5:**
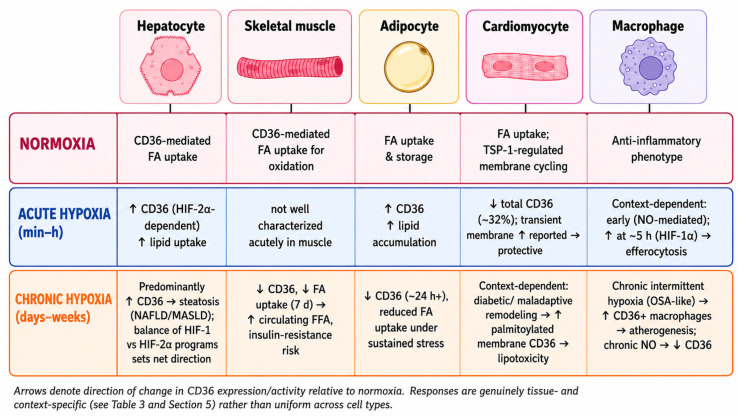
Functions of CD36 in different types of cells under physiological conditions and hypoxia. FA—fatty acids; TSP-1—thrombospondin-1. Each cell type shows a genuinely distinct, tissue-specific pattern of change across normoxia, acute hypoxia, and chronic hypoxia rather than a uniform response.

Hypoxia does not regulate CD36 uniformly; the direction and consequences of CD36 remodeling depend on the tissue type, degree, and duration of the hypoxic insult and surrounding metabolic conditions (e.g., living at high altitude, diabetes, obesity, dyslipidemia, obstructive sleep apnea, fatty liver disease, and cancer). These tissue- and time-dependent patterns are summarized in Figure 5 and Table 3.

Deciphering the role of CD36 in these circumstances (Table 4) might pave the way for new therapeutic strategies targeting CD36 expression or its multiple molecular interactions.

## 5. Tissue-Specific Implications

### 5.1. Cardiovascular Disease

In the heart, CD36 is expressed in both endothelial cells and cardiomyocytes [8]. Its physiological role is to provide cardiomyocytes with fatty acids as a major energy source. However, in pathological conditions such as ischemia, diabetic cardiomyopathy, or ischemic cardiomyopathy, CD36 is associated with adaptive and potentially maladaptive processes [8].

The effect of myocardial hypoxia on CD36 is especially context-dependent because it varies with ischemia versus hypoxia alone, reperfusion status, diabetic metabolic background, and whether the response is measured as total expression or membrane translocation.

Under hypoxic conditions (i.e., myocardial infarction), HIF-1α is activated and promotes metabolic survival pathways. In many ischemic settings, the downregulation of CD36 contributes to a metabolic shift away from fatty acid utilization and toward greater reliance on glycolysis [8]. In diabetes, the effect of HIF-1α is reduced, diminishing the adaptation to hypoxia, and this mechanism is linked to an increase in FA metabolism [60]. Consequently, diabetic hearts are less metabolically flexible under ischemic conditions and preferentially use FA rather than glucose. Mechanistically, the blunted HIF-1α response in diabetes attenuates PDK1 induction so that pyruvate dehydrogenase remains comparatively active and fatty acid oxidation is sustained; persistent CD36-mediated fatty acid influx then outstrips a less hypoxia-adapted mitochondrial compartment, linking the diabetic heart to the chronic, maladaptive arm of the framework outlined in Section 3.2 (Figure 4). Even if CD36 is downregulated under ischemia/reperfusion conditions, it is upregulated in diabetic cardiomyopathy, which leads to lipid accumulation [9]. This contributes to accelerated atherosclerosis and early vascular aging in diabetic patients [12]. Thus, inhibition of CD36 in these subjects could be beneficial by reducing lipid buildup. The use of sulfo-N-succinimidyl oleate, a FAT/CD36 inhibitor, decreases diabetic heart dysfunction following hypoxia under experimental conditions. More recently, Zhang et al. demonstrated in a postinfarction model that hypoxia directly increases CD36 palmitoylation and consequent plasma membrane localization in cardiomyocytes and that pharmacological inhibition of CD36 palmitoylation with 2-bromopalmitate reduces fatty acid uptake, decreases ceramide and diacylglycerol accumulation, and improves cardiac function after myocardial infarction [61]. This study provides direct in vivo evidence that palmitoylation-dependent trafficking, rather than total CD36 expression, drives lipotoxic injury in the hypoxic myocardium [59]. Other studies reported a significant reduction in infarct size after the administration of CD36 inhibitors, such as EP 80317 (a synthetic hexapeptide) [62] or azapeptide CP-3 [36,63]. In addition, inhibitors of CD36 translocation to the cell membrane (exenatide) improve cardiac function after ischemia–reperfusion injury [64].

In atherosclerosis, CD36 overexpression has been demonstrated to lead to lipid overload, inflammation, foam cell formation, endothelial apoptosis, macrophage trapping, and thrombosis, but CD36 deficiency causes dyslipidemia, a low degree of inflammation, and metabolic imbalance [62].

It is important to distinguish between total CD36 expression and membrane-localized CD36 expression, as the two are often regulated independently in acute hypoxia. In the myocardium, total CD36 expression is typically reduced early in ischemia, whereas membrane-localized CD36 may transiently increase by trafficking before net downregulation dominates; therefore, the direction of the observed effect depends on which pool is measured and at what time point.

Early studies revealed that acute hypoxia increases CD36 translocation to the cell membrane [36]. These data demonstrate the effect of short-term hypoxia on the inhibition of metabolic switching in ox-LDL-treated cardiomyocytes, whereas long-term hypoxia seems to negate this effect [38,39]. More recent studies have confirmed that short-term hypoxia, similar to ischemic preconditioning, has a beneficial effect and can be used as a therapeutic strategy for ox-LDL-induced cardiotoxicity [39]. The same research group confirmed the beneficial effects of short-term vs. long-term hypoxia on hyperlipidemic-affected hearts in a model using treated H9c2 cardiomyoblasts and neonatal rat ventricular cardiomyocytes [40].

Chronic hypoxic conditions (e.g., high-altitude inhabitants) induce genetic variants of PPARγ and HIF-1α, which in turn modulate CD36 expression [38]. Insulin resistance, as previously mentioned, blunts HIF-1α effects and impairs myocardial adaptation and resistance to hypoxia. This phenomenon might be especially relevant in children born and raised in chronic hypoxic conditions, such as high altitudes and cyanotic diseases, where a rise in insulin resistance during puberty induces susceptibility to cardiac dysfunction and heart failure [65,66].

Among other factors that modulate CD36 under hypoxic conditions, spexin (neuropeptide Q) is a pleiotropic peptide that was initially identified in adipocytes and was later confirmed in cardiomyocytes [67]. It is involved in FA uptake during hypoxia through the upregulation of CD36, among other factors such as carnitine palmitoyl transferase 1 (CPT1), acyl-CoA dehydrogenase medium chain (ACADM), peroxisome proliferator-activated receptor alpha (PPAR-α), and peroxisome proliferator-activated receptor-gamma coactivator 1 alpha (PGC1-α), and protects against hypoxic metabolic and mitochondrial dysfunction [67].

### 5.2. Skeletal Muscle

A recent in vitro study demonstrated that long-term (7 days) mild (4% O_2_) or severe (1% O_2_) hypoxia decreased the expression levels of FA transporters such as CD36 and solute carrier family 27 fatty acid transporter, member 4 (SCL27A4) in rat L6 differentiated myotubes, with a subsequent decrease in FA uptake. The authors concluded that this mechanism might contribute to increased levels of circulating free FA (FFA) and lead to the development of pancreatic β-cell dysfunction and insulin resistance. These hypoxia-induced dysfunctions in FFA metabolism were alleviated mostly through the upregulation of SCL27A4 expression induced by metformin treatment [68]. These findings suggest that prolonged hypoxia in skeletal muscle may impair fatty acid disposal and indirectly contribute to systemic metabolic dysfunction. Interpreted within the proposed framework, skeletal muscle illustrates the maladaptive end of the continuum under sustained hypoxia. Rather than the acute, trafficking-based restraint of fatty-acid influx seen in the myocardium, prolonged hypoxia here produces a transcriptional downregulation of CD36 that impairs local fatty-acid disposal and, by raising circulating free fatty acids, propagates lipotoxic stress to other tissues, a tissue-level adaptation that becomes systemically maladaptive.

### 5.3. Liver Disease

CD36 is an important receptor of hepatic fatty acid uptake. Intermittent hypoxia, which is seen in obstructive sleep apnea, upregulates CD36 expression and may contribute to the development of nonalcoholic fatty liver disease (NAFLD). In a recent in vitro study, Rey et al. reported that after 36 h of exposure to hypoxic conditions, both human and murine hepatocytes overexpressed HIF-1α and HIF-2α and increased lipid accumulation and CD36 overexpression through an HIF-2α-dependent mechanism [28,69]. CD36 knockdown resulted in reduced lipid accumulation under the same circumstances. Interestingly, an older in vivo study in mice revealed that in diet-induced fatty liver, HIF-1α knockout drove lipid accumulation via lipin1, a transcriptional coactivator of PPAR, and reduced CD36 expression, suggesting that HIF-1α is a key determinant of adaptive responses and prevents lipid accumulation [30]. Consistent with the tissue-specific nature of this regulation, in the kidney, HIF-2α has been reported to suppress CD36-mediated lipid accumulation in dendritic cells, thereby attenuating renal ischemia–reperfusion injury [70].

Another recent study demonstrated an additional mechanism that controls hepatic lipid accumulation through mitochondrial sirtuin 3 (SIRT3), a mitochondrial NAD-dependent deacetylase that controls mitochondrial β-oxidation. In SIRT3-deficient mice, an adaptive pathway involving increased HIF-1α and lipin1 supports PPAR-mediated fatty acid oxidation and limits hepatic lipid accumulation. However, SIRT3-deficient mice that were fed a high-fat diet, as well as SIRT3-knockdown human Huh-7 hepatoma cells incubated in palmitate, showed marked hepatic steatosis. A second mechanism revealed by this study involves increased CD36 and VLDLR expression, which promotes liver triglyceride accumulation via an Nrf2-dependent mechanism with a consequent reduction in hepatic succinate that, in turn, decreases HIF-1α hydroxylation and activation. This increase in CD36 expression was blocked in vitro by ML385, a nuclear factor erythroid 2-related factor 2 (Nrf2) inhibitor, opening new perspectives on future therapeutic approaches for NAFLD [53].

Hepatic CD36 regulation under hypoxia appears to depend on the balance between adaptive responses linked to HIF-1 and steatogenic HIF-2α- and Nrf2-linked programs. A recent mechanistic study revealed a self-reinforcing YBX1/CD36 positive feedback loop in metabolic dysfunction-associated steatotic liver disease (MASLD), in which lipid stimulation upregulated YBX1, which in turn transcriptionally increased CD36 expression; restoring CD36 expression reversed the protective effects of hepatocyte-specific YBX1 deletion [71]. This identifies an additional transcriptional amplifier in the chronic–hypoxic/chronic–lipotoxic phase of the framework proposed in Section 4. Within the three-phase framework proposed above (Section 4), this maps onto an acute, HIF-1α-dominated phase that restrains lipid accumulation and a chronic, HIF-2α- and Nrf2-driven phase that promotes CD36-mediated steatosis.

### 5.4. Tumorigenesis

Tumors provide a prototype setting in which chronic hypoxia, altered lipid availability, and immune remodeling converge, making CD36 particularly relevant to disease progression.

In a hypoxic environment, CD36 may support tumor progression not only by increasing lipid uptake by cancer cells but also by reshaping stromal, immune, and angiogenic responses within the tumor microenvironment.

Tumor cells have dysregulated, enhanced lipid metabolism to meet the requirements of rapid proliferation and development [72]. The role of CD36 in cancer has been extensively studied in relation to these changes in lipid metabolism that support tumor growth and metastasis [73]. CD36 regulates the tumor microenvironment (TME) and affects cancer progression through lipid metabolism, immune response, and angiogenesis modulation [58]. It binds to ligands such as fatty acids (FAs), cholesterol, thrombospondin-1 (TSP-1), and thrombospondin-2 (TSP-2). CD36 mediates lipid uptake in tumor-associated immune cells, cancer-associated fibroblasts (CAFs), and tumor cells, promoting tumor proliferation, invasion, and metastasis [58]. It regulates epithelial–mesenchymal transition (EMT), enhancing cancer cell migration and invasion [74]. CD36 enhances tumor-associated macrophage (TAM) polarization and recruitment, promoting tumor progression [75]. CD36 plays dual roles in angiogenesis. It inhibits angiogenesis by binding to TSP-1 and TSP-2, promoting endothelial apoptosis and antagonizing VEGF activity [76]. Conversely, CD36 can promote tumor angiogenesis through vascular mimicry (VM), where tumor cells form vascular-like structures independent of endothelial cells [77].

The balance between the pro- and antiangiogenic actions of CD36 in tumors is determined primarily by the local ligand environment and the posttranslational state of the receptor. TSP-1/TSP-2 are abundant, for example, in early stromal responses and in the CD36-positive microvasculature and bind to the antiangiogenic, proapoptotic axis through Fyn/p38/caspase-3 signaling, antagonizing VEGF. When oxidized lipids and long-chain fatty acids dominate, such as in advanced, lipid-rich tumor microenvironments, in metastasis-initiating cells, and in tumor-associated macrophages, CD36 instead engages in protumorigenic lipid acquisition and immunosuppressive signaling. Loss of TSP-1, chronic HIF-2α-driven CD36 upregulation, and palmitoylation-dependent stabilization of CD36 at the plasma membrane all shift this balance toward the proangiogenic, prometastatic state, which is consistent with the chronic-phase end of the continuum proposed in Section 4.

CD36 regulates fatty acid uptake and the balance of energy metabolism and has been shown to be upregulated in multiple cancer types, including acute myeloid leukemia, breast cancer, colorectal cancer, and gastric cancer [78]. CD36 participates in the regulation of tumor growth, metastasis, and drug resistance through diverse molecular mechanisms and is correlated with poor prognosis in various cancer types [79]. CD36 plays a protumorigenic role in breast cancer by enhancing the proliferation/migration of breast cancer cells while attenuating tamoxifen-induced inhibition of ER-positive cell growth. Moreover, CD36 has been shown to promote breast cancer metastasis, and tumor growth and enhanced expression have been correlated with increased mortality [80]. Hypoxia induces CD36 expression in gastric cancer cells and peritoneal metastasis through FA uptake [55]. The role of CD36 has been studied in other types of cancers, and it is now considered a potential biomarker and therapeutic target [81].

The tumor microenvironment is characteristically hypoxic, and hypoxia may drive CD36 upregulation in cancerous cells in a manner similar to that observed in atherosclerotic plaques [16]. Thus, CD36 plays a multifaceted role in cancer progression, influencing lipid metabolism, immune regulation, angiogenesis, and metastasis. Its diverse functions present both challenges and opportunities for developing targeted therapies. Within the framework proposed here, the tumor microenvironment represents the chronic, sustained-hypoxia extreme of the continuum (Section 4), in which durable HIF-2α-driven CD36 expression and stable membrane localization support continuous lipid acquisition rather than the transient, protective downregulation characteristic of acute hypoxia.

Initial clinical translation of CD36-directed therapy is now under way: PLT012, a humanized CD36-blocking monoclonal antibody, has demonstrated antitumor activity in preclinical models of hepatocellular carcinoma (HCC) and colorectal liver metastases by reprogramming the lipid-rich tumor microenvironment and restoring effector T-cell and NK-cell function [82] and has entered phase 1 clinical testing in patients with advanced solid tumors, with the FDA Fast Track designation granted for HCC in 2026. This first-in-class antibody approach is conceptually distinct from small-molecule CD36 inhibitors because it targets ligand binding rather than total receptor levels, illustrating a compartment-selective therapeutic strategy.

### 5.5. Brain Hypoxia

#### 5.5.1. Acute Hypoxia

In a mouse model of cerebral ischemia–reperfusion, Cho et al. demonstrated a new mechanism of CD36-mediated ROS production in ischemic brain injury. They reported that CD36 expression was upregulated in microglia/macrophages after ischemia–reperfusion. They used a model of middle cerebral artery transient occlusion (MCAO) in wild-type mice and CD36-deficient mice. The authors reported that in wild-type mice, CD36 expression was greater after brain ischemia, particularly in microglia, with a peak at 1–3 days, whereas in CD36-deficient mice, the infarct area was smaller than that in wild-type controls, and the neurological deficits were milder. Thus, Cho et al. reported that postischemic hyperemia and ROS production are decreased in CD36-null mice and proposed that the improved responses to hypoxia rely on a decrease in ROS levels during the postischemic period’s critical phase. The authors consider CD36 a potential therapeutic target for future treatments of ischemic stroke [47].

However, CD36 plays contrasting roles in the acute and resolution phases of ischemic stroke [83]. A study using a myeloid-specific HIF-1α-KO mouse model revealed that cells recovered faster after acute stroke because of decreased CD36-dependent phagocytic activity of local microglia, which led to improved neuronal survival [84]. However, after the first week post-ischemia, increased CD36 expression in infiltrating macrophages seems to be beneficial for the resolution of inflammation through increased phagocytosis during the recovery phase [56]. CD36 inhibition reduces brain injury in transient stroke (short-term hypoxia) but not in established ischemic stroke [57].

#### 5.5.2. Chronic Hypoxia

The tumor microenvironment is the best example of chronic hypoxia in the brain, which represents a key mechanism in malignant progression through the activation of HIF-1α. HIF-1α is highly expressed in glioblastoma patients but is also expressed at high levels in malignant astrocytomas, which are low-grade gliomas [85]. HIF-1α expression correlated with tumor grade, tumor progression, and invasion.

Moreover, a recent study using an animal model of glioma with Treg-specific ablation of HIF-1α revealed increased survival. In Tregs, HIF-1α promotes a metabolic shift from FA utilization toward glycolysis, thus promoting their migration over their suppression. However, under hypoxia, their suppressive function is preserved by the presence of FFAs in the tumor environment. Studies have shown that in tumors, compared with other T-cell subsets in the brain and other locations, Treg cells have higher expression of FA transporters such as CD36 [46]. Thus, the results of this study highlight potential mechanisms that could be targeted by future therapeutic strategies.

Hypoxia plays a crucial role in the onset and progression of Alzheimer’s disease by increasing oxidative stress and inflammation and inhibiting β-amyloid (Aβ) degradation. Some studies suggest that the effects of hypoxia can be mitigated by the upregulation of CD36 expression in microglia induced by NRF2 treatment, as NRF2 regulates the alternative first exons of CD36. This study demonstrated that in APP/PS1 double-transgenic mice, this treatment diminished Aβ deposition and improved spatial memory defects [86]. This study provides a promising new strategy for preventing hypoxia-induced deterioration of microglia.

### 5.6. Retinal Hypoxia

Retinal hypoxia provides a strong example of HIF-dependent CD36 regulation that overlaps with angiogenic control.

In the retina, hypoxia was proven to lead to neovascularization. However, TSP-1 binding to CD36 has been shown to block angiogenesis by attenuating VEGF signaling through VEGFR2 dephosphorylation [87].

CD36 is expressed by corneal and retinal epithelial cells [88,89]. An in vivo study by Mwaikambo et al. demonstrated that CD36 mRNA expression was markedly upregulated by hypoxia in corneal and retinal human tissues in a time-dependent manner through the activation of HIF-1, as the human CD36 promoter region has a functional binding site for HIF-1 [88]. In terms of the adaptive-to-maladaptive framework, the retina exemplifies the acute, HIF-1α-driven phase in which hypoxia-induced CD36 upregulation is coupled, through TSP-1 engagement, to an anti-angiogenic and therefore initially protective response. The transition to maladaptive, pathological neovascularization arises when sustained hypoxia shifts the ligand balance away from this TSP-1–CD36 axis toward unopposed VEGF signaling.

### 5.7. Macrophages and Inflammation

Macrophages provide some of the best examples of context-dependent CD36 regulation under hypoxia. CD36 may contribute either to inflammatory lipid loading or to resolution-phase phagocytosis depending on the timing and microenvironment.

In hypoxic environments such as inflammatory foci, HIF-1 plays a role in the adaptation of extravasated leucocytes, and HIF-1α increases bactericidal activity in activated macrophages. Diaz et al. reported that concurrent NO synthesis during the course of the inflammatory response prevents HIF-1α stabilization in macrophages, prolonging the inflammatory process and suppressing phagocytosis [21].

CD36 plays important roles in the regulation of TAM function as well as the inflammatory tumor microenvironment. This provides additional information that pharmacological inhibition of CD36 might be a promising therapy [90].

Hypoxic stimulation of macrophages also increases the expression of both thrombospondin (TSP) and CD36 in a HIF-1α-dependent manner, thus modulating macrophage phagocytosis of neutrophils. During hypoxia, blocking p38-MAPK reduces both CD36/TSP-1 expression and HIF-1α accumulation in macrophages, a pattern also observed in biopsies from patients with inflammatory bowel disease [24].

Chronic intermittent hypoxia in mice, similar to that detected in human obstructive sleep apnea (OSA), resulted in the recruitment and accumulation of highly CD36-expressing macrophages in the aorta [41]. The atherogenic process was not resolved by the interruption of chronic intermittent hypoxia but was not observed in CD36-KO mice [41]. This observation is clinically relevant, as the findings of this study demonstrate that in atherogenesis triggered by OSA, the underlying pathogenic mechanisms should be more carefully investigated, and the modulation of CD36 expression should be considered a potentially more effective therapeutic strategy. These macrophage observations map directly onto the two arms of the framework. In the acute phase, HIF-1α-dependent CD36 induction supports adaptive, resolution-oriented functions such as efferocytosis and phagocytosis of apoptotic cells, whereas under chronic intermittent hypoxia, the same CD36 induction sustains lipid-laden, CD36-high macrophage accumulation and foam-cell formation, driving the persistent, maladaptive inflammation that underlies OSA-associated atherogenesis.

## 6. Therapeutic Implications and Future Perspectives

The therapeutic direction may depend on the tissue, timing, and disease stage.

There may be an optimal protective window for CD36 expression, depending on the clinical setting. Modulation of protein function by either overexpression or deficiency, as well as modulation of CD36 function at the posttranslational level, has shown clinical potential as a possible therapeutic strategy [62]. Manipulation of CD36 in cancer therapy similarly seems to be a promising field [91]. Moreover, inhibition of CD36 in diabetic hearts might modulate myocardial metabolism to better cope with hypoxia [59]. This assumption might also be valid for populations living in chronic hypoxic conditions, such as children with cyanotic diseases or high-altitude populations. Inhibiting RCAN1 might also offer a therapeutic avenue by reducing CD36-mediated oxLDL uptake [92].

Studying the transition from acute to chronic hypoxia will be essential for identifying when CD36-targeted interventions are protective or potentially harmful and will help clarify the most clinically relevant mechanisms.

## 7. Limitations of the Current Literature

Integrating the CD36–hypoxia literature into a single framework is inherently constrained by heterogeneity in how studies are designed, reported, and interpreted. Several specific limitations deserve emphasis.

First, there is no standardized definition of acute, subacute, or chronic hypoxia across the published literature. In vitro studies variably use “acute” to describe exposures of minutes, hours, or up to 24–48 h—the latter already involving transcriptional programs and therefore arguably subacute. In vivo models add additional variability through differences in oxygen concentration (typically 1–5% O_2_ in vitro versus measured tissue pO_2_ in the low single-digit mmHg range in vivo), reperfusion timing, and concurrent nutrient availability. Direct comparison across studies is therefore often confounded by the very temporal distinction this review seeks to articulate. A further, often underappreciated, source of heterogeneity is the method used to induce hypoxia itself: chemical HIF stabilizers (e.g., cobalt chloride and dimethyloxalylglycine) bypass physiological oxygen sensing by prolyl hydroxylases and factor-inhibiting HIF altogether and can produce nonphysiological, off-target effects, whereas physical hypoxia chambers and in vivo ischemia models engage the full oxygen-sensing cascade but differ substantially from one another in terms of the rate of oxygen decline and concurrent substrate availability; studies using these different approaches are not necessarily comparable even when nominally reporting the same “% O_2_” or duration.

Second, the distinction between ischemia and pure hypoxia is frequently unclear. Ischemia restricts not only oxygen but also glucose and fatty acid delivery and impairs the clearance of metabolic waste products, whereas isolated hypoxia (as modeled in most in vitro systems) leaves the substrate supply intact. Conclusions drawn from ischemia–reperfusion models cannot be generalized to tissue hypoxia in the absence of flow restriction—for example, placental or tumor-microenvironmental hypoxia.

Third, species- and cell-type-specific differences in CD36 promoter architecture and posttranslational machinery limit the translational relevance of rodent studies. Human and murine CD36 differ in the relative usage of alternative promoters, in the regulation of palmitoylation sites, and in tissue-specific splice variant expression. Studies performed in immortalized cell lines (e.g., H9c2 and Huh-7) add a further layer of variability relative to primary cells or human tissue.

Fourth, most CD36 studies rely on bulk tissue or bulk cell analysis, which averages expression across heterogeneous populations. Single-cell and spatial transcriptomic data on CD36 expression within hypoxic microenvironments—particularly within tumors and atherosclerotic plaques—are still sparse. The consequence is that mechanistic claims at the level of specific cell subsets (e.g., TAM subpopulations, distinct microglial states, and individual trophoblast lineages) often rely on inference rather than direct measurement.

Fifth, the pharmacological tools used to probe CD36 function are imperfect. Sulfo-N-succinimidyl oleate (SSO), EP 80317, and azapeptide CP-3 each have off-target effects or incomplete selectivity, and only a subset of the “bioactive compounds” listed in Table 2 have been validated as genuinely CD36-specific in vivo. Interpretation of inhibitor studies should therefore be calibrated to this limitation. Beyond selectivity, the translational application of CD36 inhibitors is further constrained by anticipated adverse effects of systemic, tissue-nonselective CD36 modulation: human CD36 deficiency is associated with dyslipidemia, impaired oral fat tolerance, and increased susceptibility to insulin resistance, and broad CD36 blockade would be expected to impair physiological fatty acid uptake by the heart and skeletal muscle and to perturb platelet and macrophage function. Strategies that achieve tissue- or compartment-selective modulation, for example, by targeting palmitoylation, trafficking, or specific ligand-binding surfaces rather than total receptor abundance, are therefore likely to be more translationally viable than nonselective inhibitors are and represent the most realistic path to clinical application.

Sixth, much of the apparent contradiction in the CD36 literature reflects methodological rather than biological divergence. Total CD36 (measured by qPCR or Western blot) and plasma-membrane CD36 (measured by surface biotinylation or membrane fractionation) are regulated by distinct mechanisms (transcription and degradation versus vesicular trafficking and palmitoylation) and respond differently to the duration and severity of hypoxia; only the membrane pool directly mediates fatty acid uptake and ligand signaling. Studies reporting total CD36 alone cannot be directly compared with those measuring surface CD36 or functional uptake, and the choice of readout (fatty acid flux, oxLDL binding, TSP-1 signaling, or foam-cell formation) can determine whether CD36 activity appears to increase or decrease even when total expression is unchanged. Differences in oxygen tension and exposure protocol, the presence of concurrent ischemia, and the metabolic and inflammatory background (diabetes, dyslipidemia, cytokine milieu) further modulate ligand availability and posttranslational regulation. Reconciling the field will require studies that report membrane-localized CD36 and functional uptake alongside total expression under explicitly defined durations of hypoxia. Specific recurring sources of apparent disagreement include pure-hypoxia versus ischemia–reperfusion models (which combine hypoxia with substrate restriction and reperfusion-driven oxidative stress and therefore overrepresent acute CD36 internalization while underrepresenting chronic transcriptional responses); in vitro versus in vivo studies (with in vitro normoxic controls at 21% O_2_ that physiologically resemble hyperoxia and may exaggerate the magnitude of hypoxic CD36 responses); and immortalized cell lines versus primary cells or freshly isolated tissue (with cell lines often carrying altered HIF-2α or Nrf2 baselines and which may either over- or under-respond compared with primary cells). These methodological choices systematically bias the literature in directions that should be considered when interpreting individual studies. A related and frequently overlooked source of disagreement is isoform misattribution: many studies describe a “HIF-dependent” effect on CD36 using pan-HIF stabilizers or antibodies that do not distinguish HIF-1α from HIF-2α or infer isoform involvement from mRNA changes alone; without isoform-specific knockdown, overexpression, or reporter systems, it is often unclear whether an observed effect reflects HIF-1α, HIF-2α, or both, which likely contributes to the apparently conflicting tissue-specific conclusions summarized in Table 3.

Finally, the clinical translation of CD36 modulation remains preliminary. No CD36-specific drug has received regulatory approval, and the strongest translational signals come indirectly, for example, from PCSK9 inhibitors, which modulate platelet CD36 function as a secondary effect. Reviews such as the present one, therefore, necessarily synthesize predominantly preclinical literature, and the strength of the translational claims should be calibrated accordingly.

Acknowledging these limitations does not diminish the utility of the acute/chronic framework proposed here; rather, it identifies the specific methodological and conceptual refinements needed before CD36-targeted interventions can be meaningfully evaluated in clinical settings.

## 8. Conclusions

CD36 plays important roles in lipid handling and cellular adaptation under hypoxic stress. Specific tissue conditions and disease states can influence fatty acid uptake, oxidized lipid signaling, phagocytosis, angiogenesis, and cell fate. The role and regulation of CD36 appear to be connected to the duration, degree, and onset of hypoxia. In acute hypoxia, CD36 regulation may support adaptive substrate switching and limit injury, whereas in chronic hypoxia, sustained CD36 activity contributes to lipid accumulation, inflammation, tissue remodeling, and disease progression. Current studies are difficult to compare because they differ in oxygen levels, exposure duration, cell type, and disease model. Before CD36-targeted interventions are defined, future studies should first standardize experimental models that distinguish acute, subacute, and chronic hypoxia. Such clarification may support CD36 as a clinically useful target in cardiovascular disease, metabolic liver disease, cancer, and neuroinflammatory conditions. We propose that these duration-dependent effects are best understood as a single three-phase continuum: (1) acute, trafficking-driven adaptation; (2) a subacute transcriptional transition; and (3) chronic, lipotoxic remodeling, in which the balance between HIF-1α- and HIF-2α-driven programs, together with the distinction between total and membrane-localized CD36, determines whether CD36 activity is protective or pathogenic. This framework also highlights the most realistic translational opportunities. Rather than systemic suppression of total CD36, which is likely to recapitulate the adverse metabolic phenotype of human CD36 deficiency, selective interference with CD36 trafficking, palmitoylation, or specific ligand interactions offers a more tractable route. In particular, palmitoylation is a druggable posttranslational modification: the DHHC palmitoyl-acyltransferases responsible for CD36 modification and the corresponding APT/ABHD17 depalmitoylases are emerging targets, and their selective modulation could in principle reduce membrane CD36 in chronic–hypoxic lipotoxic contexts (e.g., NAFLD, diabetic cardiomyopathy, and tumor-associated macrophages) without abolishing physiological fatty acid uptake. Early translational development of a CD36-blocking antibody further supports the feasibility of context-selective targeting. Disrupting specific ligand interactions, for example, the CD36–oxLDL or CD36–TSP-1 interfaces, represents a complementary approach with greater functional specificity than receptor-level blockade does. These tissue-specific contrasts are substantial rather than incidental: acute hypoxia predominantly downregulates CD36 in the myocardium to limit lipotoxic fatty acid influx, whereas the same acute window predominantly upregulates CD36 in hepatocytes and adipocytes to increase lipid uptake, and macrophage CD36 can move in either direction depending on the inflammatory phase; chronic hypoxia most often sustains or increases CD36-mediated lipid handling in liver, tumor, and vascular macrophage compartments, driving steatosis, atherogenesis, and tumor lipid fueling, while its chronic effect on skeletal muscle is predominantly suppressive. Finally, while the acute-protective/chronic-maladaptive framework holds across most tissues, exceptions should be noted on a tissue-specific basis: in tumor-associated macrophages and in metastasis-initiating cells, sustained CD36 expression is invariably protumorigenic, whereas in efferocytic macrophages during inflammation resolution, sustained CD36 activity is required for clearance of apoptotic cells and is therefore beneficial. The therapeutic objective is therefore tissue- and context-selective modulation rather than uniform CD36 suppression or activation.

## Figures and Tables

**Table 1:** Ligands of CD36 and their effects.

CD36 Ligand	Effect
Long-chain free fatty acids	Tends to drive uptake/storage/signaling (can be adaptive or lipotoxic) [13].
oxLDL/oxidized phospholipids	tends to connect hypoxia/inflammation to foam cell-like lipid loading and inflammatory signaling (but may be downregulated in some hypoxic plaque settings where LOX-1 dominates) [16]
Thrombospondin-1	Tends to drive anti-angiogenic signaling and endothelial apoptosis [15]

**Table 2:** Pharmacological modulation of CD36 [19].

Pharmacological Modifiers	Target Effect	Main Effect
**Drugs**		
Statins	Suppress CD36 expression	Via PPARγ-dependent pathways in macrophages and vascular smooth muscle cells [19,25]
Nifedipine	Reduces CD36 expression	Reduces lipid uptake in macrophages [19]
Ezetimibe:	Decreases CD36 expression	Decreases foam cell formation [19]
Tamoxifen	Inhibits CD36 expression	Inhibits ox-LDL accumulation in macrophages [19]
**Bioactive Compounds**		
Andrographolide	Promotes CD36 degradation	Reduces foam cell formation [19]
Pomegranate Peel Polyphenols	Suppress CD36 expression	Reduce lipid uptake [19]
Spiromastixones	Decrease CD36 expression	Promote cholesterol efflux [19]
Puerarin	Reduces CD36 expression	Foam cell formation [19]
Tanshinone IIA	Targets platelet CD36	Reduces platelet activation [19]
Orientin	Inhibits ox-LDL-induced CD36 expression	Reduces inflammatory responses [19]
Quercetin	Reduces CD36 expression	Reduces foam cell formation [19,26]
Curcumin	Modulates CD36 expression	Modulates fatty acid metabolism [19]

**Table 3:** Tissue-specific effects of acute and chronic hypoxia on CD36 expression and function.

Tissue/Key Cell Type(s)	Effect of Chronic/Long-Term Exposure to Hypoxia (Days → Weeks)	Effect of Acute/Short-Term Exposure to Hypoxia (Minutes → Hours)
**Myocardium** **(cardiomyocytes)**	Downregulation of CD36—long-term energy depletion in chronic ischemia [38].	Typically, downregulation of CD36 (~32%) limits fatty acid uptake and protects against ischemia/reperfusion injury [8]. However, in some acute hypoxia or preconditioning models, CD36 upregulation and metabolic switching toward fatty acid utilization have been reported, depending on context (e.g., oxLDL exposure) [39,40]
**Vascular wall** **(aortic macrophages)**	Increased accumulation of CD36+ macrophages in the aorta during chronic intermittent ischemia (obstructive sleep apnea) promoting atherogenesis [41].	Increased oxLDL uptake and early subclinical atherosclerosis associated with obstructive sleep apnea [42].
**Pulmonary vascular wall (lung tunica media cells)**	Upregulation of CD36 in pulmonary vascular smooth muscle cells under chronic hypoxia [43].	—
**Adipose tissue** **(adipocytes)**	Decreased CD36 expression after ~24 h hypoxia, suggesting reduced fatty acid uptake under sustained stress [44].	Upregulation of CD36 expression and enhanced lipid accumulation during early hypoxic exposure [28].
**Macrophages**	CD36 is also downregulated by nitric oxide in inflammatory hypoxic environments, while iNOS inhibition may increase CD36 expression [21]. Decreased CD36 expression after 24 h hypoxia, independent of HIF-1α [16].	In early inflammation, CD36 is often downregulated [21]. During the resolution phase (~5 h hypoxia), HIF-1α upregulates CD36 and promotes phagocytosis of apoptotic cells [24].
**Liver (hepatocytes)**	In chronic hypoxia and NAFLD models, HIF-1-dependent pathways may reduce CD36 expression and limit lipid accumulation [30].	Upregulation of CD36 expression and enhanced lipid accumulation during early hypoxia [28].
**Brain (glioblastoma cells; microglia)**	CD36 upregulation in glioblastoma and other chronically hypoxic tumor environments [45,46].	Upregulation in microglia following ischemia–reperfusion injury [47].
**Retinal cells**	Upregulation of CD36, modulated by HIF-1α, in 24 h hypoxic medium [48].	—

Abbreviations: HIF-1α, hypoxia-inducible factor 1 alpha; iNOS, inducible nitric oxide synthase; NAFLD, nonalcoholic fatty liver disease; oxLDL, oxidized low-density lipoprotein; h, hour(s).

**Table 4:** Mechanistic differences in CD36 regulation and function in acute and chronic hypoxia.

Feature	Effect of Chronic/Long-Term Exposure to Hypoxia	Effect of Acute/Short-Term Exposure to Hypoxia
**Time scale**	Sustained low O_2_ exposure with persistent activation of transcriptional programs (e.g., HIF-driven responses) [27,31].	Rapid-onset energy stress with immediate signaling responses (e.g., AMPK activation, ROS changes) [31,52].
**Dominant control of CD36 impact**	Programmatic regulation: transcriptional control (HIF-dependent and independent), posttranslational modifications (e.g., palmitoylation), and sustained membrane localization [34,50].	Rapid regulation: trafficking and recycling between intracellular compartments and the plasma membrane, with limited early transcriptional contribution [34,36].
**CD36 localization & lipid influx**	Often increased or sustained CD36 surface localization, promoting long-chain fatty acid and/or oxLDL uptake (strongly context- and tissue-dependent) [16,28,55].	Frequently transient reduction in surface CD36 (e.g., in ischemic cardiomyocytes), limiting fatty acid uptake; however, some models show increased CD36 translocation depending on metabolic context [39,52].
**Metabolic consequence**	Potential mismatch between continued lipid influx and reduced oxidative capacity → lipid droplet accumulation, DAG/ceramide production, mitochondrial stress, and lipotoxicity [34,53].	Shift toward glycolysis with reduced reliance on fatty acid oxidation; limiting fatty acid influx may decrease formation of toxic lipid intermediates during acute stress [39,40].
**Inflammation/repair functions**	Sustained CD36–ligand signaling may promote chronic inflammation, foam cell formation, fibrosis, and tissue remodeling; receptor dominance may shift (e.g., toward LOX-1 in atherosclerosis) [19,24].	Context-dependent: CD36 may be downregulated during early inflammation but upregulated during resolution to enhance efferocytosis (e.g., CD36–TSP-1 axis) [56,57].
**Typical net tendency**	Increased risk of lipotoxicity, chronic inflammation, and progressive tissue dysfunction, depending on disease context (e.g., NAFLD, atherosclerosis, tumors) [28,38,58].	Often adaptive in the short term, aligning substrate use with reduced oxygen availability; outcomes depend on tissue type and reperfusion dynamics [39,59].

Abbreviations: AMPK—AMP-activated protein kinase; DAG—diacylglycerol; HIF—hypoxia-inducible factor; LOX-1—lectin-like oxidized LDL receptor-1; O_2_—oxygen; oxLDL, oxidized low-density lipoprotein; ROS—reactive oxygen species; TSP-1—thrombospondin-1.

## Data Availability

No new data were created or analyzed in this study. Data sharing is not applicable to this article.

## Abbreviations

The following abbreviations are used in this manuscript:

Aβ	amyloid beta
ACADM	acyl-CoA dehydrogenase, medium chain
AdipoR2	adiponectin receptor 2
AMPK	AMP-activated protein kinase
APP/PS1	amyloid precursor protein/presenilin 1
ATF2	activating transcription factor 2
ATP	adenosine triphosphate
CAFs	cancer-associated fibroblasts
CECs	circulating endothelial cells
CD36	cluster of differentiation 36
CPT1	carnitine palmitoyltransferase 1
DAG	diacylglycerol
EMT	epithelial–mesenchymal transition
ER	estrogen receptor
FA	fatty acid
FAO	fatty acid oxidation
FAT	fatty acid translocase
FFA	free fatty acid
GLU	glucose
GLUT1	glucose transporter 1
GLUT4	glucose transporter 4
GP IV	glycoprotein IV
HCC	hepatocellular carcinoma
HIF	hypoxia-inducible factor
HIF-1α	hypoxia-inducible factor 1 alpha
HIF-2α	hypoxia-inducible factor 2 alpha
iNOS	inducible nitric oxide synthase
IRGM1	immunity-related GTPase family M member 1
I/R	ischemia–reperfusion
KO	knockout
LCFA	long-chain fatty acid
LCFAs	long-chain fatty acids
LOX-1	lectin-like oxidized low-density lipoprotein receptor 1
MAPK	mitogen-activated protein kinase
MCAO	middle cerebral artery occlusion
miR-181a	microRNA-181a
NAFLD	nonalcoholic fatty liver disease
Nrf2	nuclear factor erythroid 2-related factor 2
NO	nitric oxide
O_2_	oxygen
OSA	obstructive sleep apnea
oxLDL	oxidized low-density lipoprotein
PAH	pulmonary arterial hypertension
PDH	pyruvate dehydrogenase
PDK1	pyruvate dehydrogenase kinase 1
PGC1-α	peroxisome proliferator-activated receptor gamma coactivator 1 alpha
PHD	prolyl hydroxylase domain
PKCθ	protein kinase C theta
PLIN2	perilipin 2
PPAR-α	peroxisome proliferator-activated receptor alpha
PPAR-γ	peroxisome proliferator-activated receptor gamma
PTM	posttranslational modification
PTMs	posttranslational modifications
PXR	pregnane X receptor
RCAN1	regulator of calcineurin 1
ROS	reactive oxygen species
SIRT3	sirtuin 3
SLC27A4	solute carrier family 27 member 4
Src	Src family kinase
SR-B2	scavenger receptor B2
STAT5	signal transducer and activator of transcription 5
TAMs	tumor-associated macrophages
TME	tumor microenvironment
Treg/Tregs	regulatory T cell(s)
TSP	thrombospondin
TSP-1	thrombospondin-1
TSP-2	thrombospondin-2
VEGF	vascular endothelial growth factor
VEGFR2	vascular endothelial growth factor receptor 2
VLDLR	very low-density lipoprotein receptor
VM	vascular mimicry
